# Short-and long-latency afferent inhibition of the human leg motor cortex by H-reflex subthreshold electrical stimulation at the popliteal fossa

**DOI:** 10.1007/s00221-022-06497-2

**Published:** 2022-12-08

**Authors:** Tatsuya Kato, Atsushi Sasaki, Kimitaka Nakazawa

**Affiliations:** 1 Graduate School of Arts and Sciences, Department of Life Sciences, The University of Tokyo, 153-8902 Tokyo, Japan; 2 Japan Society for the Promotion of Science, Tokyo, 102-0083 Japan; 3 Graduate School of Engineering Science, Department of Mechanical Science and Bioengineering, Osaka University, Osaka, 560-8531 Japan

**Keywords:** Short-latency afferent inhibition, Long-latency afferent inhibition, Transcranial magnetic stimulation, Hoffman reflex, Sensorimotor integration

## Abstract

In humans, peripheral sensory stimulation inhibits subsequent motor evoked potentials (MEPs) induced by transcranial magnetic stimulation; this process is referred to as short- or long-latency afferent inhibition (SAI or LAI, respectively), depending on the inter-stimulus interval (ISI) length. Although upper limb SAI and LAI have been well studied, lower limb SAI and LAI remain under-investigated. Here, we examined the time course of the soleus (SOL) muscle MEP following electrical tibial nerve (TN) stimulation at the popliteal fossa at ISIs of 20–220 ms. When the conditioning stimulus intensity was three-fold the perceptual threshold, MEP amplitudes were inhibited at an ISI of 220 ms, but not at shorter ISIs. TN stimulation just below the Hoffman (H)-reflex threshold intensity inhibited MEP amplitudes at ISIs of 30, 35, 100, 180 and 200 ms. However, the relationship between MEP inhibition and the P30 latency of somatosensory evoked potentials (SEPs) did not show corresponding ISIs at the SEP P30 latency that maximizes MEP inhibition. To clarify whether the site of afferent-induced MEP inhibition occurs at the cortical or spinal level, we examined the time course of SOL H-reflex following TN stimulation. H-reflex amplitudes were not significantly inhibited at ISIs where MEP inhibition occurred but at an ISI of 120 ms. Our findings indicate that stronger peripheral sensory stimulation is required for lower limb than for upper limb SAI and LAI and that lower limb SAI and LAI are of cortical origin. Moreover, the direct pathway from the periphery to the primary motor cortex may contribute to lower limb SAI.

## Introduction

The integration of peripheral afferent information and motor commands is essential for motor control (Rothwell et al. 1982; Wiesendanger and Miles 1982). In humans, paired-pulse paradigms have been used to explore how sensory signals modulate the primary motor cortex (M1). Specifically, peripheral sensory stimulation inhibits subsequent motor evoked potentials (MEPs) induced by transcranial magnetic stimulation (TMS); this process is referred to as short-latency afferent inhibition (SAI) and long-latency afferent inhibition (LAI) (Sailer et al. 2003). SAI refers to afferent inhibition with an inter-stimulus interval (ISI) of 20–50 ms, which is mainly related to the afferent conduction time of peripheral sensory stimulation, while LAI refers to inhibition with ISIs after 100 ms (Chen et al. 1999; Tokimura et al. 2000; Abbruzzese et al. 2001; Tamburin et al. 2003; Kessler et al. 2005; Helmich et al. 2005; Fischer and Orth 2011). SAI and LAI are considered of cortical origin because F-waves following peripheral sensory stimulation were found to be unchanged (Chen et al. 1999; Voller et al. 2006). Both SAI and LAI involve a cholinergic pathway, the activity of which is modulated by GABA_A_ (Di Lazzaro et al. 2005, 2007; Turco et al. 2018a). SAI and LAI can be practical tools for evaluating sensorimotor function and disease or injury (Sailer et al. 2003; Di Lazzaro et al. 2012; Cengiz et al. 2019). Furthermore, recent studies have reported changes in SAI and LAI during or after motor and cognitive tasks (Bonnì et al. 2017; Mineo et al. 2018; Mirdamadi and Block 2020).

However, studies on SAI and LAI of the lower limbs are lacking. Unlike the upper limb, the human lower limb is involved in locomotion and postural stability associated with various movements. In other words, it is the basis of standing movements. Decreased sensorimotor function of the lower limbs results in gait and postural instability (Dingwell and Cusumano 2000; Menz et al. 2004; Belda-Lois et al. 2011). Therefore, methods for assessing lower limb sensorimotor function are essential. However, lower limb SAI and LAI have not been sufficiently studied. For the upper limbs, previous studies have suggested that the ISIs of SAI and LAI are related to the afferent conduction time of peripheral afferents to the cortex (Chen et al. 1999; Kessler et al. 2005; Kotb et al. 2005; Fischer and Orth 2011). MEPs are most inhibited at ISIs slightly longer than the N20 component latency of somatosensory evoked potentials (SEPs), which are recorded as responses on electroencephalography (EEG) after peripheral nerve stimulation (Kessler et al. 2005; Fischer and Orth 2011). LAI of the upper limbs occurs 100–200 ms after peripheral sensory stimulation (Chen et al. 1999; Kotb et al. 2005), which is close to the time at which the bilateral secondary somatosensory (S2) and contralateral posterior parietal (PPC) cortices are activated (Hari et al. 1984; Forss et al. 1994; Korvenoja et al. 1999; Waberski et al. 2002). However, the pathway of LAI remains unknown because afferent information can reach various cortical areas 100 ms after peripheral sensory stimulation (Allison et al. 1989, 1992). In contrast to the upper limb, the ISIs and conditioning stimulus intensities required for lower limb SAI and LAI are uncertain. Previous studies have demonstrated that MEPs of the resting soleus (SOL) muscle following tibial nerve (TN) stimulation at motor threshold (MT) stimulus intensity were not significantly inhibited at ISIs of 20–100 ms, but were facilitated at ISIs of 45–60 ms (Poon et al. 2008; Roy and Gorassini 2008). Nevertheless, a previous study targeting the tibial anterior (TA) muscle reported that MEPs were inhibited at ISIs of 45–80 ms when a train of three consecutive pulses at three-fold the perceptual threshold (PT) was applied to the dorsal surface of the foot (Ruddy et al. 2016). When TN stimulation with 1.5-fold the MT was applied below the malleolus, TA MEPs were inhibited at ISIs of 32.5–37.5 ms and SOL MEPs were also inhibited (ISIs were not shown) (Roy and Gorassini 2008). Furthermore, to our knowledge, studies on lower limb LAI are lacking.

Therefore, this study aimed to explore the ISIs and stimulus intensities of the peripheral sensory stimulation required to induce MEP inhibition in lower limb muscles. To this end, we investigated the time course of lower limb muscle MEPs following peripheral sensory stimulation at the popliteal fossa with several intensities at both short and long ISIs. The Hoffman reflex (H-reflex) was also assessed using the same protocol to explore spinal modulation by peripheral sensory stimulation. We assumed that H-reflexes following peripheral sensory stimulation would not change at any ISI when the stimulus intensity was below the H-reflex threshold because of a very small postsynaptic effect. Furthermore, for short ISIs, we examined the relationship between SEP latency and the degree of MEP inhibition to reveal the association between the afferent conduction time of peripheral afferents to the cortex and MEP inhibition by peripheral stimulation. This study would provide insight into the sensory information flow from the lower limb to M1.

## Methods

### Participants

A total of 21 healthy volunteers (19 male, 2 female; aged 26.0 ± 3.1 years) participated in four experiments. Five volunteers participated in all experiments. We confirmed that none of the participants had previous experience with convulsions or seizures, metal implants, neurostimulators, cardiac pacemakers, intracardiac lines, or history of neurological diseases such as epilepsy, and they did not regularly use medications such as antidepressants or other neuromodulatory drugs (Rossi et al. 2021). All participants provided written informed consent, and the study was conducted in accordance with the tenets of the Declaration of Helsinki. The experimental procedures were approved by the local institutional ethics committee of the corresponding author.

### Electromyography

Surface electromyography (EMG) was performed on the right SOL muscle using surface Ag/AgCl electrodes (Vitrode F-150-S, Nihon Kohden, Japan). EMG signals were pre-amplified 1000-fold and filtered with a bandpass filter of 5–1000 Hz (MEG-6108, Nihon Kohden, Japan). All EMG signals were digitized at 4000 Hz using an analog-to-digital converter (Power lab/16SP, AD Instruments, Australia) and stored on a computer for offline analysis.

### TMS

A conditioning test paradigm was used to investigate lower limb SAI and LAI. Single-pulse monophasic TMS for test stimulation (TS_TMS_) was applied over the left (contralateral) M1 with a posterior-anterior current direction using a double-cone coil connected to a Magstim 200 stimulator (Magstim 200, Magstim Co., Whitland, UK). The optimal stimulation site (i.e., “hotspot”) for the right SOL muscle was identified to elicit MEP responses effectively and marked with a TMS tracking system (Brainsight, Rogue Research, Montreal, QC, Canada) to confirm the placement of the coil. The TS_TMS_ intensity was adjusted to evoke SOL MEPs of approximately 0.1 mV (Roy and Gorassini 2008).

### Peripheral nerve stimulation

**Fig. 1:**
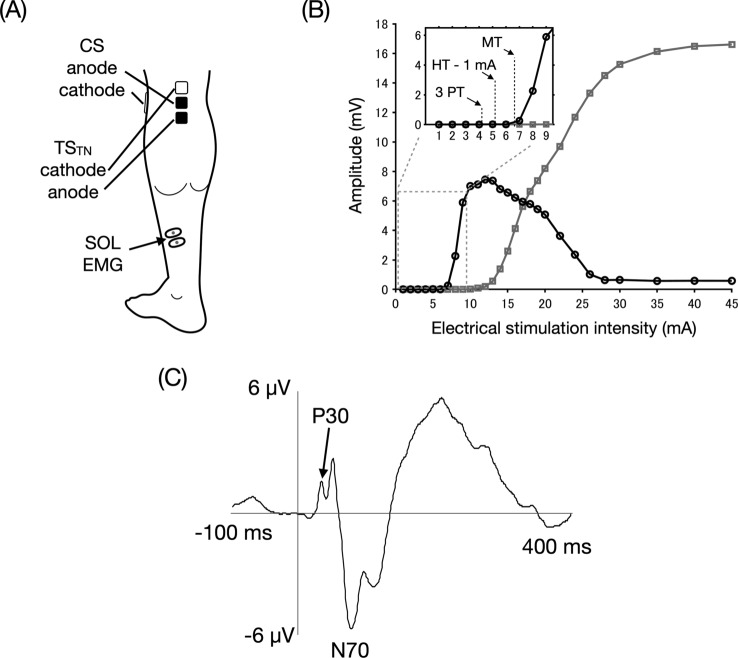
Experimental setup and somatosensory evoked potential waveform. **A** Electrodes for conditioning and test tibial nerve (TN) stimulation (CS and TS_TN_, the black and white are the anode and cathode, respectively) and for electromyography (EMG) recording. **B** The H-reflex and M-wave recruitment curves (a black line and circles: H-reflex, a gray line and squares: M-wave). *PT* perceptual threshold, *HT* H-reflex threshold, *MT* motor threshold **C** an averaged waveform of somatosensory evoked potentials following TN stimulation in a representative participant. P30 appeared first. Following N70, negative and positive peaks appeared at approximately 100 and 200 ms after stimulation, respectively

Conditioning TN stimulation (CS) was performed using a constant-current electrical stimulator (Digitimer, DS7A, Hertford, UK). The anode (5 × 5 cm) was placed over the patella, and the cathode (2 × 2 cm) was positioned at the popliteal fossa where the SOL muscle H-reflex could be induced most effectively (Fig. 1A). The pulse width was set to 1 ms. The CS intensity was set to three-fold the PT in Experiment 1 and the H-reflex threshold (HT) minus 1 mA in Experiments 2, 3 and 4 to keep the number of α-motoneurons in the refractory period low and minimize the postsynaptic effect. The H-reflex and M-wave recruitment curves were obtained from one participant to clarify the nature of the CS (Fig. 1B). Test TN stimulation (TS_TN_) was applied with a constant-current electrical stimulator (Digitimer, DS7R) to examine spinal modulation by the CS. The anode (2 × 2 cm) was placed 1 cm below the cathode for the CS, and the cathode (2 × 2 cm) was placed above the cathode for the CS, at which point the SOL muscle H-reflex could be induced most effectively (Poon et al. 2008; Roy and Gorassini 2008) (Fig. 1A). The pulse width was set to 1 ms. The TS_TN_ intensity was adjusted to evoke 50% of the maximum H-reflex response to observe both inhibitory and facilitatory modulation equally (Crone et al. 1990). These stimulation protocols have been established in previous studies and allow for stable electrical stimulation throughout the experiment (Stein et al. 2007; Poon et al. 2008; Roy and Gorassini 2008; Kumpulainen et al. 2012; Andrews et al. 2020).

### SEPs

Eleven participants were tested (nine male, two female; aged 25.3 ± 3.0 years). SEPs evoked by TN stimulation at the knee were recorded using a 64-channel EEG cap with electrodes arranged in the international 10–20 electrode system (Waveguard original, ANT Neuro b.v., Enschede, the Netherlands) and a mobile amplifier (eego sports, ANT Neuro b.v.). The reference and ground electrodes were placed on CPz and AFz, respectively. The anode and cathode for TN stimulation were placed at the same locations as those for the CS. A total of 360 stimuli were delivered via surface electrodes at a rate of 1.98 Hz in three 1 min blocks with a constant-current electrical stimulator (Digitimer, DS7A). The stimulus intensity was set at 5% of the maximal M-wave recorded in the SOL muscle (Wasaka et al. 2006). EEG signals were digitized at 2000 Hz and resampled at 1000 Hz before the analysis. Then signals were filtered at 1–100 Hz using a noncausal finite impulse response bandpass filter (Widmann et al. 2015). After noise removal from blinks, eye movements, and neck movements using independent component analysis, the EEG signals were segmented into epochs from 100 ms prior to 400 ms after the TN stimulation. The value within 50 ms prior to stimulation was subtracted from epoch signals for baseline correction, and thereafter, epochs exceeding ± 100 µV were rejected. Approximately 350 EEG signals were averaged. The lower limb SEP was obtained from the Cz electrode based on previous studies (Wasaka et al. 2006; Peters et al. 2019; Kato et al. 2022). The latency of the first positive peak (P30) of the SEPs was measured (Fig. 1C).

### Experiment 1: time course of SOL MEPs following TN stimulation

Fourteen subjects (all male; aged 26.6 ± 3.5 years) participated in this experiment. The participants were seated with their knee joints at 45° using a footrest to allow effective stimulation of the TN. To investigate the time course of SOL MEPs following TN stimulation, we tested 10 ISIs (20, 25, 30, 35, 40, 45, 50, 100, 200 and 220 ms). ISIs of 20–50 ms were selected for SAI to account for the first P30 SEP peak (Fig. 1C). ISIs of 100, 200 and 220 ms were selected for LAI based on previous reports that ISIs of 100–200 ms induce consistent inhibition of MEPs in the upper limbs (Chen et al. 1999; Kotb et al. 2005; Turco et al. 2017) and different conduction times from the periphery to the cortex in the upper and lower limbs. The CS intensity was set at 3PT (3.0 ± 1.3 mA), which has been demonstrated to be sufficiently strong to induce upper limb SAI by median nerve stimulation (Ni et al. 2011). Moreover, a previous study reported that CS with 3PT intensity on the dorsal surface of the foot inhibited MEPs of the TA muscle (Ruddy et al. 2016). To establish the baseline MEP amplitude, eight MEPs were obtained using TS_TMS_ alone at the beginning of the experiment. Eight paired stimuli (CS and TS_TMS_) at each ISI were then delivered in a random ISI order. The reason for the small number of TMS pulses was to avoid inducing changes in corticospinal excitability due to paired stimulation effects during the experiment despite including various ISIs (Mrachacz-Kersting et al. 2007; Roy et al. 2007). At the end of the experiment, to confirm that SOL corticospinal excitability remained constant throughout the experiment, eight MEPs were obtained with TS_TMS_ alone.

### Experiment 2: effects of stronger TN stimulation on SOL MEP

Sixteen subjects (14 male, 2 female; aged 26.3 ± 3.2 years) participated in this experiment. A previous study used the MT for the CS intensity and observed SAI of the SOL muscle in some participants (Roy and Gorassini 2008). Comparing Experiment 1 of the present study with their results suggests that a stronger intensity is more effective in modulating SOL MEPs with TN stimulation at the knee. However, since TN stimulation at the MT intensity was sufficiently strong to elicit SOL H-reflex (Poon et al. 2008) (Fig. 1B), the CS of intensity eliciting H-reflex may inhibit MEPs at the spinal level but not at the cortical level owing to the postsynaptic effect (Matthews 1996; Kiernan et al. 2004; Poon et al. 2008). Therefore, we used the HT minus 1 mA for CS intensity (8.1 ± 4.0 mA; 87.0 ± 5.6% of the HT) to minimize the number of α-motoneurons in the refractory period due to the CS. Moreover, the HT minus 1 mA was higher than 3PT in all participants except one (3.0 ± 1.4 mA). The experimental setup and protocol were identical to those used in Experiment 1.

### Experiment 3: time course of SOL H-reflexes following TN stimulation

Thirteen subjects (11 male, 2 female; aged 25.8 ± 3.3 years) participated in this experiment. Using the same CS intensity as in Experiment 2 (8.0 ± 4.2 mA; 86.7 ± 5.9% of the HT), we investigated the effects of conditioning TN stimulation on spinal excitability to reveal whether MEP inhibition by peripheral sensory stimulation is of spinal or cortical origin. The experimental protocol was the same as in Experiment 2, with the use of TS_TN_ instead of TS_TMS_.

### Experiment 4: modulation of SOL MEPs and H-reflexes following TN stimulation at long ISIs

Twelve subjects (ten male, two female; aged 26.8 ± 3.4 years) participated in this experiment. Previous studies examining upper limb SAI and LAI have reported MEP inhibition even at ISIs between 60 and 200 ms (Tamburin et al. 2001; Kotb et al. 2005). This experiment aimed to closely examine the modulation of corticospinal and spinal excitability with paired stimulation at long ISIs. TS_TMS_ or TS_TN_ was applied after the CS at nine ISIs (60, 70, 80, 90, 100, 120, 150, 180 and 200 ms) in different blocks. The CS intensity was set to the HT minus 1 mA (7.5 ± 3.9 mA; 85.8 ± 6.0% of the HT). The other experimental protocols were the same as those in Experiments 2 and 3. Eight MEPs were obtained using TS_TMS_ or TS_TN_ alone at the beginning of the block, the paired stimulation paradigm was performed at nine ISIs, and eight MEPs were obtained using TS_TMS_ or TS_TN_ alone again at the end of the block. After the first block, the second block was conducted using the other test stimulation.

### Data analysis

The size of the MEP and H-reflex responses were measured as the peak-to-peak amplitudes. Statistical comparisons of MEP and H-reflex amplitudes were performed with normalized data, which are expressed as a percentage of responses produced by TS alone before paired stimulation. Values < 100% indicate inhibition, and values > 100% indicate facilitation by CS.

**Fig. 2:**
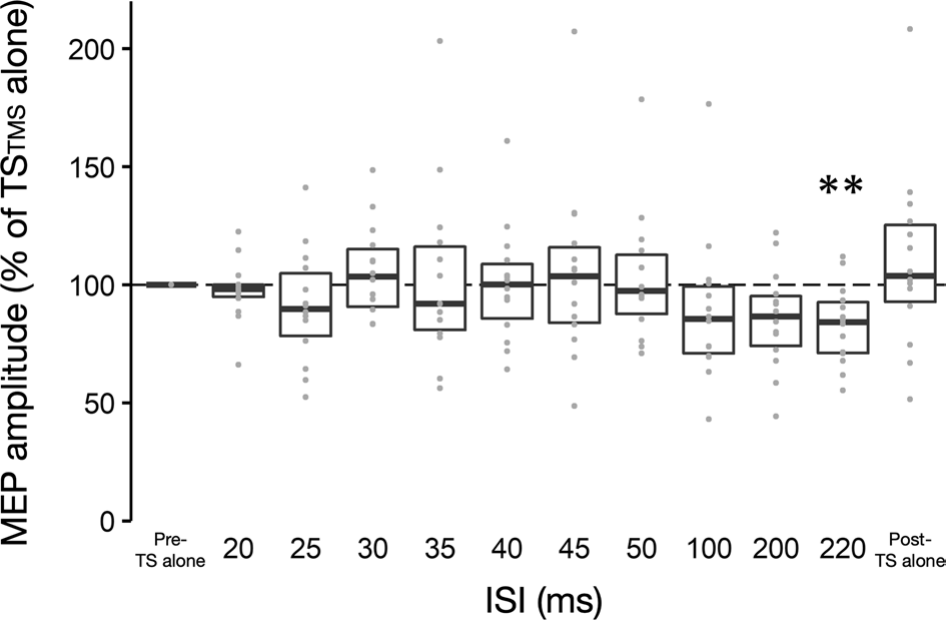
Time course of soleus muscle motor evoked potentials following conditioning tibial nerve stimulation with three-fold the perceptual threshold intensity at inter-stimulus intervals of 20–220 ms. The baseline is the mean motor evoked potential (MEP) amplitude with test transcranial magnetic stimulation (TS_TMS_) alone (Pre-TS alone) and is shown as a black dotted line. Conditioned MEP amplitudes and MEP amplitude with TS_TMS_ alone after paired stimulation (Post-TS alone) are normalized by the baseline. The boxplot shows group data, and the gray dots show individual participant data. ** *P* < 0.01 compared to baseline. *ISI* inter-stimulus interval

All statistical analyses were performed using R software (version 4.0.5, R Foundation for Statistical Computing, Vienna, Austria). The Shapiro–Wilk test was performed to examine whether the data were normally distributed. The time course of the MEP and H-reflex amplitudes was assessed using the Friedman test of variance (analysis of variance) with ISI as a within-subject factor in Experiments 1–4, as the data at some ISIs were non-normally distributed (e.g., in Experiment 1, the ISI of 50 ms: *P* = 0.03; Fig. 2). The Wilcoxon signed-rank test (two-tailed) with Bonferroni correction for multiple comparisons was used as a post hoc test to test for significant changes at different ISIs compared to TS alone. Note that TS alone after paired stimulation was included in the statistical model above to test for differences in TS alone before and after paired stimulation. In Experiment 2, we also examined the relationship between the SEP P30 latency and MEP inhibition. For participants who completed the SEP experiment, MEPs at ISIs of 20, 25, 30, 35 and 40 ms were converted to MEPs at P30 + x ms ISI using the individual SEP P30 latency. After the normalized MEP data were log-transformed, second-order polynomial regression was performed. The reason why a second-order polynomial model was chosen is that we hypothesized that MEP inhibition would be the greatest at a certain ISI relative to the SEP P30 latency, similar to previous studies on upper limb SAI (Kessler et al. 2005; Fischer and Orth 2011). An *F* test comparing a second-order polynomial model and a constant function was used to examine the validity of the model fitting result. Kendall’s *W* and *r* values were calculated as the effect size in the Friedman and Wilcoxon signed-rank tests, respectively (0 ≤ *W, r* ≤ 1, *W* = 0 and *r* = 0 indicate no relationship). The significance level was set at *P* < 0.05.

## Results

### Experiment 1: time course of SOL MEPs following TN stimulation

Figure 2 presents the time course of SOL MEPs following TN stimulation (CS) with 3PT intensity at ISIs of 20–220 ms. The size of SOL MEP amplitudes was modulated depending on the ISI (*P* = 0.005, *W* = 0.18). Post-hoc analysis revealed that conditioned SOL MEP amplitudes at ISIs of 220 ms were significantly reduced compared to baseline (83 ± 17%, *P* = 0.007, *r* = 0.65). There was no significant difference in SOL MEP amplitudes of TS_TMS_ alone before (i.e., baseline) and after (110 ± 38%, *P* = 1, *r* = 0.26) paired stimulation, indicating that there were no changes in SOL corticospinal excitability throughout the experiment.

### Experiment 2: effects of stronger TN stimulation on SOL MEP

**Fig. 3:**
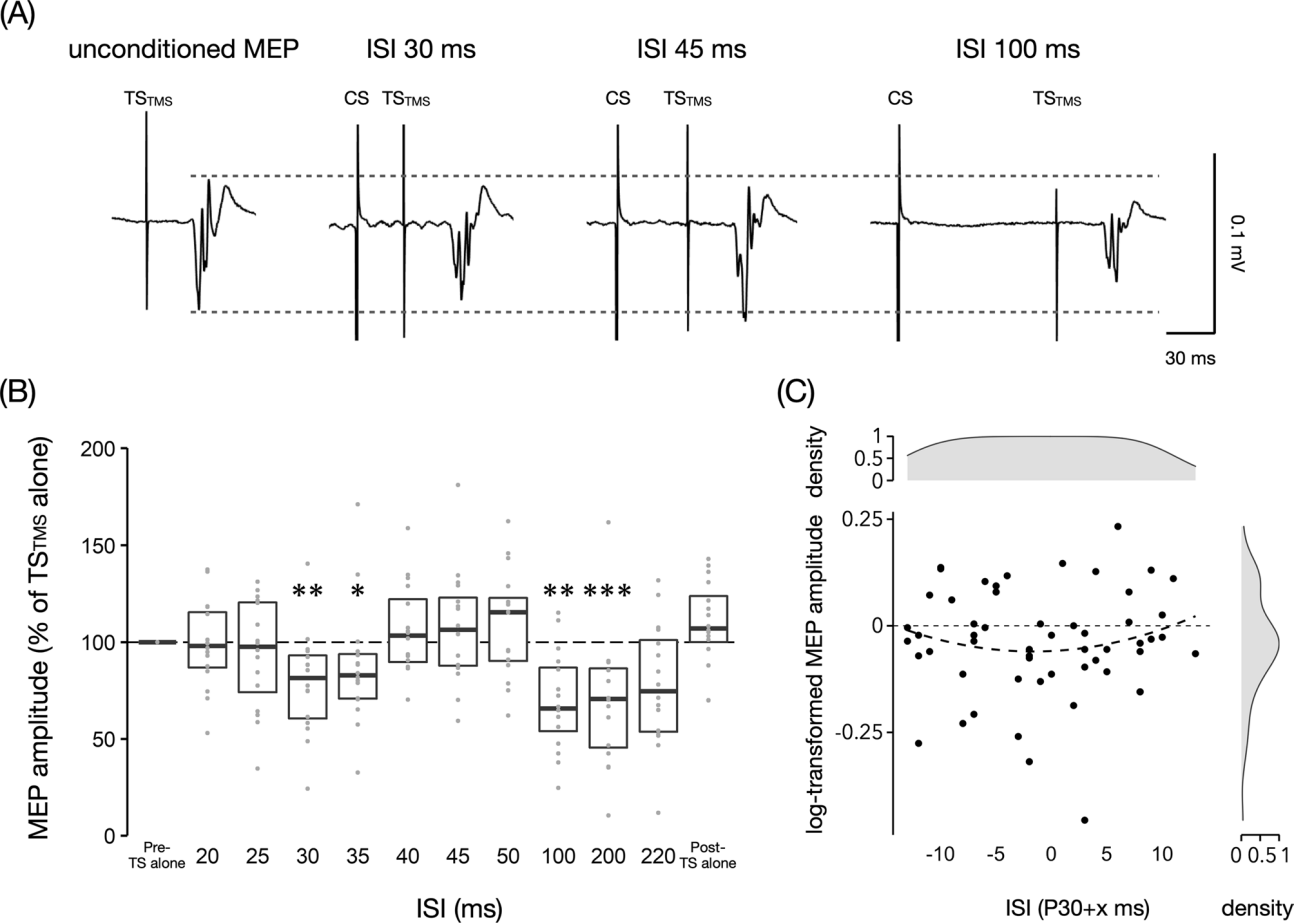
Soleus motor evoked potentials following conditioning tibial nerve stimulation with the H-reflex threshold minus 1 mA intensity. **A** Examples of mean soleus (SOL) muscle motor evoked potentials (MEPs) following conditioning tibial nerve (TN) stimulation (CS) in a representative participant with test transcranial magnetic stimulation (TS_TSM_) at inter-stimulus intervals (ISIs) of 30, 45, and 100 ms. **B** Time course of SOL MEPs following CS with the H-reflex threshold minus 1 mA intensity at ISIs of 20–220 ms. The baseline is the mean MEP amplitude with TS_TMS_ alone (Pre-TS alone) and is shown as a black dotted line. Conditioned MEP amplitudes and MEP amplitude with TS_TMS_ alone after paired stimulation (Post-TS alone) are normalized by the baseline. The boxplot shows group data, and the gray dots show individual participant data. **C** A second-order polynomial trend between ISIs relative to the somatosensory evoked potential P30 latency and MEP modulation is shown as a dark gray dotted curve with a minimum at P30–1.7 ms. The ISI and MEP distributions are shown as a density plot with the maximum value being one. SOL MEPs following TN stimulation were the least inhibited at an ISI of approximately 5 ms after P30 latency. * *P* < 0.05, ** *P* < 0.01, *** *P* < 0.001 compared to baseline

Figure 3A illustrates the effect of TN stimulation (CS) with HT minus 1 mA on SOL MEPs at ISIs of 30, 45, and 100 ms. The time course of conditioned SOL MEPs is shown in Fig. 3B. The Friedman test revealed a significant effect of ISI on SOL MEP amplitudes (*P* < 0.001, *W* = 0.32). Post-hoc analysis revealed that conditioned SOL MEP amplitudes were significantly reduced compared to baseline at ISIs of 30 (79 ± 26%, *P* = 0.001, *r* = 0.68), 35 (87 ± 31%, *P* = 0.015, *r* = 0.57), 100 (69 ± 26%, *P* = 0.001, *r* = 0.68), and 200 ms (69 ± 34%, *P* < 0.001, *r* = 0.80). There were no significant differences in SOL MEP amplitudes of TS_TMS_ alone before (i.e., baseline) and after (109 ± 22%, *P* = 0.12, *r* = 0.46) paired stimulation, indicating that there were no changes in SOL corticospinal excitability throughout the experiment.

To examine the relationship between ISIs with respect to the SEP P30 latency and MEP inhibition at ISIs of 20, 25, 30, 35 and 40 ms, a second-order polynomial trend line was fitted to conditioned SOL MEP amplitudes (Fig. 3C). A downward convex quadratic function with a minimum at an ISI of P30–1.7 ms [95% confidence interval: −8.1 to 4.7] was approximated (fitted curve: *Y* = −0.00039(X + 1.7)^2^−0.06). However, the model fitting result was not statistically significant compared with that of a constant function (*F*_(2,52)_ = 1.31, *P* = 0.54) and the coefficient of determination was small (*R*^2^ = 0.023).

### Experiment 3: time course of the SOL H-reflexes following TN stimulation

**Fig. 4:**
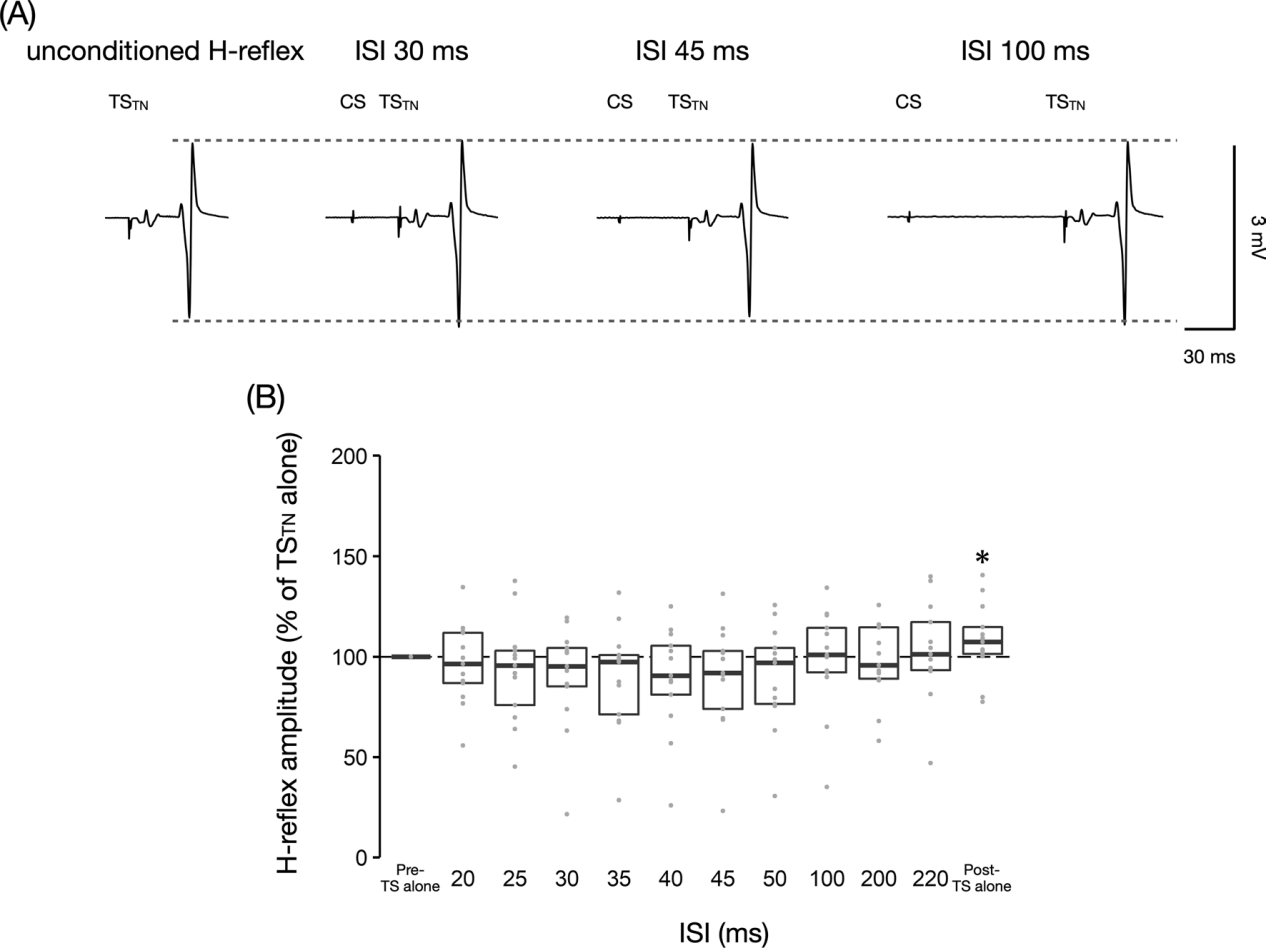
Time course of the soleus muscle H-reflex following conditioning tibial nerve stimulation with the H-reflex threshold minus 1 mA intensity at inter-stimulus intervals of 20–220 ms. **A** Examples of mean soleus (SOL) muscle H-reflex following conditioning tibial nerve (TN) stimulation (CS) in a representative participant with test TN stimulation (TS_TN_) at inter-stimulus intervals (ISIs) of 30, 45, and 100 ms. **B** Time course of SOL H-reflex following CS with the H-reflex threshold minus 1 mA intensity at ISIs of 20–220 ms. The baseline is the mean H-reflex amplitude with test tibial nerve stimulation (TS_TN_) alone (Pre-TS alone) and is shown as a black dotted line. Conditioned H-reflex amplitudes and H-reflex amplitude with TS_TN_ alone after paired stimulation (Post-TS alone) are normalized by the baseline. The boxplot shows group data, and the gray dots show individual participant data. * *P* < 0.05 compared to baseline. *ISI* inter-stimulus interval

To clarify whether the MEP inhibition observed in Experiment 2 occurred at the cortical or spinal level, we examined the time course of SOL H-reflexes following TN stimulation (CS) with HT minus 1 mA intensity at ISIs of 20–220 ms (Fig. 4). The Friedman test revealed a significant effect of ISI on SOL H-reflex amplitudes (*P* = 0.007, *W* = 0.18). Post-hoc analysis revealed that none of conditioned SOL H-reflex amplitudes was significantly reduced at any ISI (all *P* > 0.87). However, there was a significant difference in SOL H-reflex amplitudes of TS_TN_ alone before (i.e., baseline) and after (106 ± 18%, *P* = 0.016, *r* = 0.63) paired stimulation, suggesting the facilitation of SOL spinal excitability throughout the experiment.

### Experiment 4: modulation of SOL MEPs and H-reflexes following TN stimulation at long ISIs

**Fig. 5:**
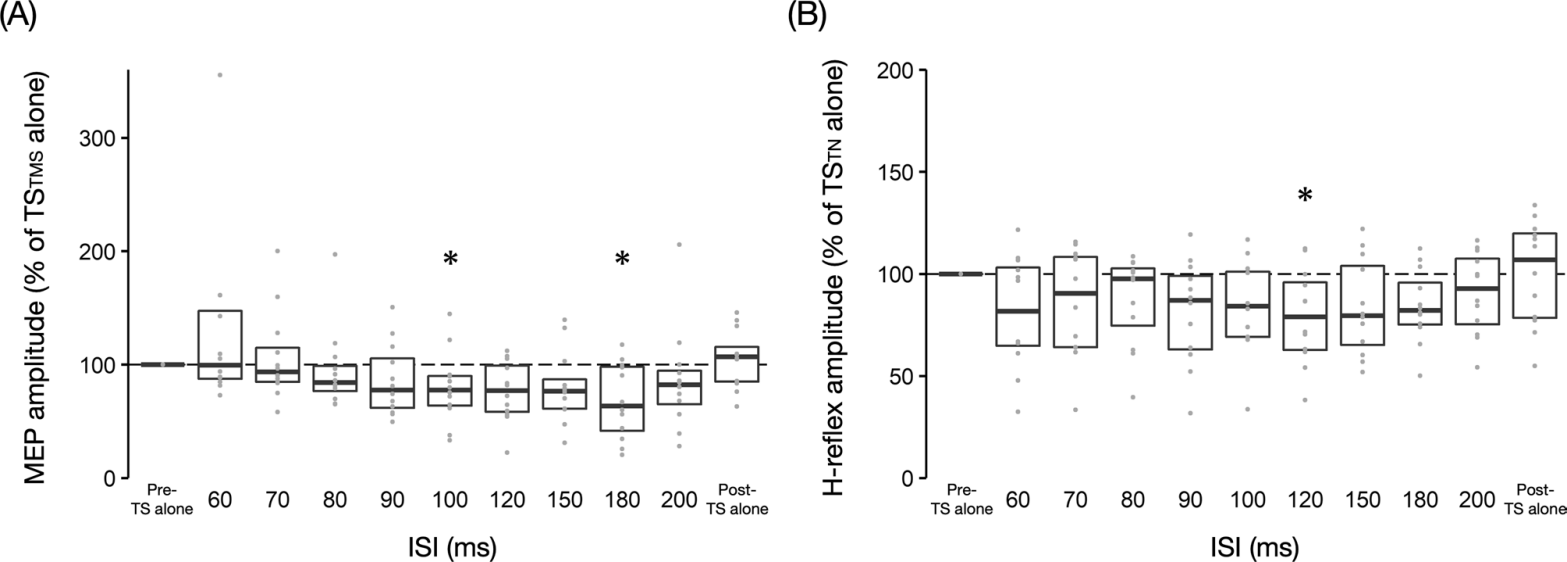
Modulation of soleus muscle motor evoked potentials and H-reflexes following conditioning tibial nerve stimulation at long inter-stimulus intervals. **A** and **B** Time course of soleus muscle motor evoked potentials (MEPs) and H-reflex, respectively, following conditioning tibial nerve (TN) stimulation with the H-reflex threshold minus 1 mA intensity at inter-stimulus intervals (ISIs) of 60–200 ms. The baseline is the mean MEP with test transcranial magnetic stimulation (TS_TMS_) alone (**A**) or the mean H-reflex amplitude with test tibial nerve stimulation (TS_TN_) alone (**B**) (Pre-TS alone) and is shown as a black dotted line. Conditioned amplitudes and the amplitude with TS alone after paired stimulation (Post-TS alone) are normalized by the baseline. The boxplot shows group data, and the gray dots show individual participant data. * *P* < 0.05 compared to baseline

The time course of SOL MEPs following TN stimulation (CS) with the HT minus 1 mA intensity at ISIs of 60–200 ms is presented in Fig. 5A. The Friedman test indicated a significant effect of ISI on SOL MEP amplitudes (*P* < 0.001, *W* = 0.40). Post-hoc analysis revealed that conditioned SOL MEP amplitudes were significantly reduced compared to baseline at ISIs of 100 (79 ± 31%, *P* = 0.034, *r* = 0.61) and 180 ms (68 ± 33%, *P* = 0.034, *r* = 0.61). There were no significant differences in SOL MEP amplitudes of TS_TMS_ alone before (i.e., baseline) and after (105 ± 25%, *P* = 1, *r* = 0.30) paired stimulation, indicating that no changes in SOL corticospinal excitability were observed throughout the experiment.

The time course of conditioned SOL H-reflexes following TN stimulation (CS) with the HT minus 1 mA intensity at ISIs of 60–200 ms is illustrated in Fig. 5B. The Friedman test showed a significant effect of ISI on conditioned SOL H-reflex amplitudes (*P* = 0.014, *W* = 0.18). Post-hoc analysis revealed that conditioned SOL H-reflex amplitudes were significantly reduced compared to baseline at an ISI of 120 ms (79 ± 23%, *P* = 0.034, *r* = 0.61). There was no significant difference in the SOL H-reflex amplitudes of TS_TN_ alone before (i.e., baseline) and after (100 ± 26%, *P* = 1, *r* = 0.15) paired stimulation, indicating no changes in SOL spinal excitability throughout the experiment.

## Discussion

We examined the time course of lower limb muscle MEPs and H-reflexes following peripheral sensory stimulation. Our results showed that SOL MEP amplitudes following TN stimulation were inhibited at both short and long ISIs. However, MEP inhibition in a wide range of ISIs was observed when the intensity of peripheral sensory stimulation was HT minus 1 mA, but not when it was 3PT. SOL H-reflex amplitudes following TN stimulation were not inhibited at ISIs at which MEP inhibition occurred but at an ISI of 120 ms. Our data indicate that lower limb SAI and LAI occurred at the cortical level, as the stimulus intensity of peripheral electrical stimulation was adjusted to minimize postsynaptic effects, which is identical to upper limb SAI and LAI (Chen et al. 1999; Tokimura et al. 2000; Voller et al. 2006).

### Lower limb SAI

One of the aims of this study was to explore the ISI of lower limb SAI. We found that SOL MEP amplitudes following TN stimulation with the HT minus 1 mA intensity were inhibited at ISIs of 30 and 35 ms. These results are similar to those of a previous study showing that SAI of the SOL muscle occurred at ISIs of 25–30 ms with the CS at the MT intensity (Roy and Gorassini 2008). However, these previous results were from an additional experiment with only five participants who exhibited MEP inhibition at an ISI of 30 ms when ISIs of 20–100 ms were employed. Further, no significant MEP inhibition at an ISI of 30 ms was observed when eight participants were evaluated with ISIs of 20–100 ms (Roy and Gorassini 2008). We confirmed that SAI of the SOL muscle stimulated at the popliteal fossa occurs at ISI of 30 ms and extended previous results by showing that it also occurs at an ISI of 35 ms. However, CS with 3PT intensity at the knee did not induce SOL muscle SAI. It has been demonstrated that upper limb SAI plateaus at 3PT intensity (Ni et al. 2011). TA MEPs were inhibited by a train of three consecutive pulses at 3PT intensity to the TN at the dorsal surface of the foot (Ruddy et al. 2016). Together, SAI by stimulation at the knee may require a higher stimulus intensity compared to stimulation at the wrist or dorsal surface of the foot. The TN descends the popliteal fossa anterior to the deep fascia and is thus located deeper in the skin than nerves at the wrist or the TN at the dorsal surface of the foot (Standring 2021). This might affect the stimulus intensity of the CS. Furthermore, the lower limbs contribute to locomotion and postural control rather than dexterous movements. The intracortical inhibitory circuits, which are modulated by GABA_A_, assist the corticospinal system in producing segmented activity in the intrinsic hand muscles (Zoghi et al. 2003). The low demand for dexterous movements in the lower limbs may result in no SAI with peripheral electrical stimulation at 3PT intensity.

Previous studies have demonstrated that median nerve stimulation inhibits MEP amplitudes at ISIs of 0–4 ms longer than the SEP N20 latency, which is the first peak of the SEP elicited by median nerve stimulation (Kessler et al. 2005; Fischer and Orth 2011). When the TN is stimulated at the knee, the first SEP peak appears at approximately 31 ms and is called P30 (Kato et al. 2022). As MEPs following TN stimulation at the knee were inhibited at ISIs of 30 and 35 ms in the present study, our results demonstrated that lower limb SAI was prominent at ISIs around the first SEP peak as well as upper limb SAI. Note that an ISI of 30 ms was faster than SEP P30 latency. Another previous study reported that when TN stimulation with 1.5-fold the MT was applied below the malleolus, TA MEPs were inhibited by TN stimulation with 1.5-fold the MT at ISIs of 32.5–37.5 ms, which are faster than afferent inputs reaching the primary somatosensory cortex (S1) from below the malleolus (Roy and Gorassini 2008). These results indicate the possibility that the sensory input pathways that inhibit MEP amplitudes differ between upper- and lower limb SAI. M1 receives sensory signals from S1 (Sakamoto et al. 1987; Kaneko et al. 1994) and directly through the thalamus (Asanuma et al. 1979; Lemon and van der Burg 1979). Previous studies have suggested that the intracortical projection time from S1 to M1 is 5–6 ms (Stinear and Hornby 2005; Mrachacz-Kersting et al. 2007). Therefore, we speculate that lower limb SAI at ISIs of 30 and 35 ms is attributed to the direct projection of somatosensory stimuli to M1 through the thalamus rather than projections of those via S1. A previous study reported loss of SAI in a patient with paramedian thalamic infarction while the SEP N20 component was intact (Oliviero et al. 2005). In contrast, another study reported loss of the SEP N20 component in a patient with posterolateral thalamus infarction while SAI was intact (Alaydin et al. 2021). These findings suggest that the thalamus has a pathway to M1 that regulates SAI, in addition to a pathway to S1. It should be noted that these previous studies focused on the upper limbs. Furthermore, we could not find a clear relationship between MEP inhibition and ISIs relative to the SEP P30 latency. Therefore, both the pathway from the thalamus directly to M1 and the pathway from the thalamus to M1 via S1 are likely involved in lower limb SAI. Given that the ISI of upper limb SAI is the SEP N20 latency plus 0–4 ms (Kessler et al. 2005; Fischer and Orth 2011), the inhibition mechanisms may differ between upper- and lower limb SAI. This may reflect differential temporal constraints on corrective action induced by peripheral sensory information between the upper and lower limbs owing to differences in functional roles.

Previous studies have reported SOL MEP facilitation when TN stimulation with MT intensity is applied 40–60 ms before TMS (Poon et al. 2008; Roy and Gorassini 2008). In contrast, we found no facilitation effect on the SOL MEP following TN stimulation. These contrasting findings may have resulted from the stimulus intensity of peripheral nerve stimulation. In the upper limb, the higher the peripheral stimulation intensity, the greater the MEP facilitation (Fischer and Orth 2011). Therefore, peripheral nerve stimulation at the stimulus intensity of the HT or higher may be effective in facilitating corticospinal excitability of lower limb muscles.

Paired associative stimulation (PAS), in which TMS and peripheral nerve stimulation are applied continuously in pairs, has been attracting attention as a non-invasive method to induce plastic changes in cortical and/or spinal excitability (Mrachacz-Kersting et al. 2007; Roy et al. 2007). For instance, after 5 min of PAS intervention (peripheral nerve stimulation at 1.5-fold MT), SOL MEP amplitudes were reduced when the ISI was the SEP P30 latency plus 6 ms (approximately 35 ms) but increased when the ISI was the SEP P30 latency plus 18 ms (approximately 50 ms). Therefore, our results indicate that the relationship between ISIs and MEP modulation could contribute to the development of neural interventions, such as PAS.

### Lower limb LAI

To the best of our knowledge, this is the first report of lower limb LAI. Specifically, strong inhibition of SOL MEPs was observed at ISIs of 100 ms and around 200 ms in this study. The ISIs are similar to those reported in previous studies on the upper limb (Chen et al. 1999; Kotb et al. 2005; Turco et al. 2017). Since SEP peaks elicited by TN stimulation at the knee appeared at approximately 100 and 200 ms, these SEP peaks may be related to lower limb LAI (Fig. 1C). The neural pathway of LAI remains unknown, mainly because afferent inputs can spread across broad cortical areas at long ISIs between peripheral sensory stimulation and TMS pulses (Turco et al. 2018b). Given that S2 and the PPC are activated 100–200 ms after electrical stimulation of the median or peroneal nerve (Allison et al. 1989; Forss et al. 1994), activities of S2 and PPC are candidates for producing LAI (Chen et al. 1999; Kotb et al. 2005; Turco et al. 2018b). However, other areas may be also involved in LAI because afferent information can reach various cortical areas 100 ms after peripheral sensory stimulation (Allison et al. 1989, 1992). For ISIs of > 220 ms, Chen et al. (1999) demonstrated that right median nerve stimulation reduces MEP amplitudes of the right-hand muscles at ISIs of 200–1000 ms; thus, there is a possibility that lower limb LAI is induced at ISIs of > 220 ms. As with SAI, when the peripheral stimulation intensity was higher than 3PT, MEP amplitudes were inhibited to a greater extent. This is in line with a previous study that demonstrated that stimulation of hand nerves at an intensity stronger than 3ST induced greater LAI of the hand muscle (Turco et al. 2017).

### H-reflex following peripheral sensory stimulation

Our results showed that SOL H-reflex amplitudes following TN stimulation were not inhibited at ISIs in which MEP inhibition occurred, suggesting that the inhibition of SOL MEPs following TN stimulation occurs via cortical mechanisms. However, the inhibition of SOL H-reflex was observed at an ISI of 120 ms in this study. Moreover, it is worth noting that in several participants, SOL H-reflex amplitudes were inhibited by the preceding TN stimulation over a wide range of ISIs (Figs. 4 and 5B). Even though the CS intensity used in this study was subthreshold, it is likely that postsynaptic effects, such as homosynaptic depression and afterhyperpolarization, were significant in the participants (Hultborn et al. 1996; Matthews 1996; Kiernan et al. 2004; Poon et al. 2008). Another explanation for this H-reflex inhibition is the oligosynaptic pathway. It has been suggested that H-reflex is not solely monosynaptic (Burke et al. 1984). The CS may activate interneurons in the oligosynaptic pathway of H-reflex, resulting in inhibition of signal transmission to α-motoneurons by the TS. Therefore, although no participants who exhibited strong H-reflex inhibition exhibited strong MEP inhibition, cortical inhibition in lower limb SAI and LAI may have been overestimated in some participants.

### Limitations

There are several limitations of this study that must be considered. First, we used the resting SOL muscle, which can easily elicit H-reflex, whereas previous studies stimulated the TA or SOL muscle at the knee or the dorsal surface of the foot with 3PT, MT, or 1.5-fold MT stimulus intensities (Poon et al. 2008; Roy and Gorassini 2008; Ruddy et al. 2016). The ISIs at which SAI occurred varied among these studies. Therefore, the reproducibility of lower limb SAI should be tested by unifying the stimulation points, muscles, and parameters. Moreover, in comparing the TA and SOL muscles, the TA muscle has a stronger corticospinal input (Morita et al. 2000). Specifically, the H-reflex and MEP amplitude of the SOL muscle increased by the same extent with voluntary plantarflexion, whereas the MEP amplitude of the TA muscle increased more with voluntary dorsiflexion compared to the H-reflex amplitude (Morita et al. 2000). Thus, the effects of tasks, interventions, and training on SAI may differ between the TA and SOL muscles. This may be one of the reasons why SOL spinal, but not corticospinal, excitability was facilitated after several combinations of peripheral sensory stimulation and TMS. Second, a second-order polynomial model implied that MEP inhibition was maximized at an ISI 1.7 ms before the SEP P30 latency. However, the coefficient of determination was small. Further studies are needed to elucidate the relationship between the ISI and SEP P30 latency in lower limb SAI. Third, we were unable to reproduce lower limb LAI at an ISI of 200 ms in Experiment 4. A previous study reported larger inter-participant variability in LAI than in SAI (Turco et al. 2019). Therefore, a larger sample may be necessary to obtain reliable LAI results. Finally, an intensity of the HT minus 1 mA was used as the CS intensity. The difference in the absolute values of the HT was not normalized across individual participants. However, the HT minus 1 mA intensity was within 80–90% of the HT in almost all participants, and there was no correlation between absolute stimulation intensities and MEP inhibition. Measuring recruitment curves would reveal the relationship between the CS intensity and lower limb SAI and LAI.

## Conclusions

In summary, the present study demonstrated that both lower limb SAI and LAI were induced when the intensity of peripheral sensory stimulation was the HT minus 1 mA, but not when it was 3PT. Since H-reflex amplitudes were not inhibited at ISIs at which MEP inhibition occurred, the lower limb SAI and LAI may be of cortical origin. Furthermore, the direct pathway from the periphery to the primary motor cortex may contribute to lower limb SAI. Our results provide a guideline to explore sensorimotor integration of the lower limbs and have the potential to be applied to the development of neural intervention methods.

In terms of the functional significance of SAI and LAI, recent studies have reported that changes in upper limb SAI and LAI are related to a maze tracing task (Mirdamadi and Block 2020), recognition memory tasks (Bonnì et al. 2017; Mineo et al. 2018; Suzuki and Meehan 2018), and a visual attention task (Mirdamadi et al. 2017). Meanwhile, a basketball shooting exercise and finger force control have been shown to be not correlated with upper limb SAI (Deveci et al. 2020; Paparella et al. 2020). It remains unclear what types of motor and cognitive functions are associated with upper limb SAI and LAI (Turco et al. 2021). Future studies to investigate the relationship between motor/cognitive functions and SAI/LAI of both the upper and lower limbs are needed to reveal the functional significance of SAI/LAI and the differences between upper- and lower limb SAI/LAI.

## Data Availability

The data that support the findings of this study are available on reasonable request from the corresponding author.

## Abbreviations

CS: Conditioning stimulation
EEG: Electroencephalography
EMG: Electromyography
H-reflex: Hoffman reflex
HT: Hoffman reflex threshold
ISI: Inter-stimulus interval
LAI: Long-latency afferent inhibition
M1: Primary motor cortex
MEP: Motor evoked potential
MT: Motor threshold
PAS: Paired associative stimulation
PPC: Posterior parietal cortex
PT: Perceptual threshold
S1: Primary somatosensory cortex
S2: Secondary somatosensory cortex
SAI: Short-latency afferent inhibition
SEP: Somatosensory evoked potential
SOL: Soleus
TA: Tibial anterior
TMS: Transcranial magnetic stimulation
TN: Tibial nerve
TS: Test stimulation
TS_TMS_: Transcranial magnetic stimulation for test stimuli
TS_TN_: Tibial nerve stimulation for test stimuli
